# Prebiotic Aggregates (Tissues) Emerging from Reaction–Diffusion: Formation Time, Configuration Entropy and Optimal Spatial Dimension

**DOI:** 10.3390/e24010124

**Published:** 2022-01-14

**Authors:** Juan Cesar Flores

**Affiliations:** Facultad de Ciencias, Universidad de Tarapacá, Casilla 7-D, Arica 1000000, Chile; cflores@uta.cl

**Keywords:** reaction–diffusion systems, origin of life, configuration entropy, fractal dimensions

## Abstract

For the formation of a proto-tissue, rather than a protocell, the use of reactant dynamics in a finite spatial region is considered. The framework is established on the basic concepts of replication, diversity, and heredity. Heredity, in the sense of the continuity of information and alike traits, is characterized by the number of equivalent patterns conferring viability against selection processes. In the case of structural parameters and the diffusion coefficient of ribonucleic acid, the formation time ranges between a few years to some decades, depending on the spatial dimension (fractional or not). As long as equivalent patterns exist, the configuration entropy of proto-tissues can be defined and used as a practical tool. Consequently, the maximal diversity and weak fluctuations, for which proto-tissues can develop, occur at the spatial dimension 2.5.

## 1. Introduction

The origin of life was a chemical event and life on Earth began some billions of years ago [1,2] and, what is more, the definition of life presents an intricate question [3,4]. Nevertheless, the interpretation of life [4] as a self-supporting chemical system capable of Darwinian evolution stands as a well-suited framework.

The present article considers a pathway for attaining elementary prebiotic processes from a non-equilibrium point of view [5]. Life is an out-of-equilibrium phenomenon, and it is, thus, natural to use reaction-diffusion equations in its study [6]. Additionally, amino acids exist in meteorites [7,8]; consequently, no emphasis is given to a particular type of reactive element at this first stage of research. The present work emphasizes the generation of not an isolated cell, but a structure (i.e., a proto-tissue) comprising elementary cells. This is the framework of complex systems and morphogenesis concepts [9,10]. Meanwhile, the relevant concept of heredity, in the sense of viability through adaptation in open systems, will be included.

The following concepts [11] are adopted as a framework and used in constructing a model of proto-tissue formation in a finite spatial region:

- Replication: Reactants interact to produce a compound A, mainly from another chemical compound, a substrate B. Metabolism is assumed implicitly in the replication process. This replication process retains a sense of order, which will be measured in terms of configuration entropy.
- Variation: Spatial patterns (containing “bricks” A) have multiple and equivalent forms for fixed values of physical and chemical parameters. This is an analog to the concept of the degeneration of states in physics [12,13,14,15].
- Heredity: on a large scale of perturbations, there is a viable continuity of equivalent traits (patterns) promoting adaptation.

Statements (a) and (b) are connected to physical and chemical procedures, where compound A corresponds to the morphogen. Statement (c) is a necessary condition of living systems [11].

This work is conducted using mathematic and simulation tools; nevertheless, qualitative explanations are offered regularly. Analytic or computational models for environments and prebiotic processes are broadly mentioned and, in this sense, this approach must be considered as a partial contribution to the following complex problem: prebiotic organization and configuration. The substrate where the aggregate of protocells develops is a simple compound, but it is enough to substantiate the notions developed in this work. I emphasize, this work considers only a lineal analysis related to possible final structures (proto-tissues). Explicit solutions and time evolution towards final structures will be performed elsewhere.

## 2. Schnakenberg’s Model and Homogeneous Solution Instability

For simplicity and to maintain basic central ideas, a generic Schnakenberg [16] reaction–diffusion equation, involving a few chemical compounds, is considered [17]. Consider two compounds in a given spatial region with densities A and B, such as in the following:

(1) $$\left[\frac{\partial }{\partial t}-D_{A}\nabla^{2}+k_{A}\right]A=+k_{AB}Af\left(A,B\right)$$

(2) $$\left[\frac{\partial }{\partial t}-D_{B}\nabla^{2}+k_{B}\right]B=-k_{AB}Af\left(A,B\right)$$

where the positive function f(A,B) corresponds to a chemical reaction between compounds. Usually, for a larger number of reacting compounds, a high-order polynomial function f(A,B) becomes useful in maintaining a two-variable model (involving slave variables and adiabatic elimination [18]). The k parameters are the usual chemical rates and the D parameters are diffusion coefficients. Boundary conditions are chosen as A=0 and B=constant at borders.

As part of the required metabolism, and for simplicity, waste is considered through the loss rate $-k_{A}$. A third explicit equation for waste does not contribute appreciably to better comprehension at this stage.

Moreover, the basic spatial solutions of the above equations are assumed as A=0 (null solution) and $B=B_{o}$ (constant). In addition, the condition $k_{B}=0$ is assumed hereafter. This homogeneous solution corresponds to the absence of structures (proto-tissues).

A boundary, a spatial (hyper-) cube, is assumed for mathematical convenience. As usual in reaction–diffusion equations [19,20,21,22,23,24,25,26,27,28,29,30], the stability of the background solution is related to the perturbation functions $A=0+\varepsilon_{o}exp\left(\lambda t+i\vec{k}\cdot \vec{x}\right)$ and $B=B_{o}+\eta_{o}exp\left(\lambda t+i\vec{k}\cdot \vec{x}\right)$. Algebraic equations for the stability parameter λ are then obtained from Equations (1) and (2):

(3) $$\lambda \varepsilon_{o}=-D_{A}{\vec{k}}^{2}\varepsilon_{o}-k_{A}\varepsilon_{o}+k_{AB}f\left(0,B_{o}\right)\varepsilon_{o}$$

(4) $$\;\lambda \eta_{o}=-D_{B}{\vec{k}}^{2}\eta_{o}-k_{AB}f\left(0,B_{o}\right)\varepsilon_{o}$$

Here, the wave vector $\vec{k}\propto \left(n,l,m\right)$ (integers) defines a typical wavelength $2\pi /\left|\vec{k}\right|$. The instability [19,20,21,22,23,24,25,26,27,28,29,30] of the homogenous solution, correlated with proto-tissue creation, is analyzed in the following sections.

Finally, long-range spatial or temporal (memory) effects [18,19,20,22,23,24] can be further considered in aggregates of protocell formation. Neural models require this kind of mathematical contribution. Particularly, the characteristic time formation (Section 6) can be improved by considering an integrodifferential contribution in Equations (1) and (2). In the same way, aspects such as cross-diffusion, where the diffusion coefficient is a tensor, or nonlinear features, can also improve the model presented in this work.

## 3. Necessary Conditions for Generating Proto-Tissues Structures

At this stage, for the generation of structures, I assume the realization of the fundamental condition (see Equation (3)) as the following:

(5) $$k_{AB}f\left(0,B_{o}\right)>k_{A}$$

which is necessary, but not sufficient, to produce instabilities. A critical phase can be defined around the equality of Equation (5). A detailed revision can ultimately be conducted on criticality in biological systems [31].

Equation (5) reveals that the rate of production of compound A is higher than its destruction. It states that morphogen A can eventually grow from a zero value through (unspecified) fluctuations.

Finally, semi-analytical solutions of Schnakenberg’s equation, including limit cycles, can be viewed in reference to Noufaey [32], where Equation (5) is, or is not, verified to obtain patterns.

## 4. Unstable Manifold: Tissue Formation

**Figure 1:**
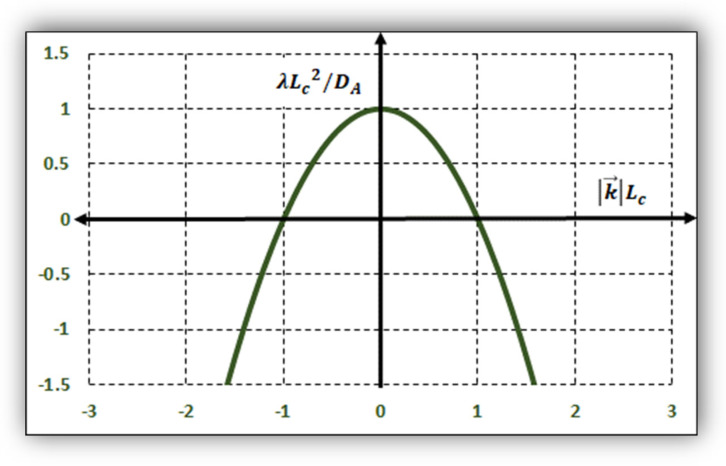
Stability dimensionless parameter $\lambda L_{c}{}^{2}/D_{A}$ as a function of the normalized wavenumber $\left|\vec{k}\right|L_{c}$. The upper region between 0 and 1, verifying Equation (5), corresponds to the unstable manifold, and, consequently, patterns can develop from A=0 (i.e., tissues). The parameter $L_{c}=\sqrt{D_{A}/\left(k_{AB}f\left(0,B_{o}\right)-k_{A}\right)}$. is the characteristic size of a protocell (Equation (9) with α=1).

From the reaction–diffusion stability, Equations (3) and (4) become an eigenvalue problem [19,20,21,22,23,24,25,26,27,28,29,30] associated with the following algebraic equation:

(6) $$\left(D_{B}{\vec{k}}^{2}+\lambda \right)\left(k_{AB}f\left(0,B_{o}\right)-D_{A}{\vec{k}}^{2}-k_{A}-\lambda \right)=0$$

the first manifold $\lambda_{st}=-D_{B}{\vec{k}}^{2}$ is always stable. No structures are produced in this mode. The second manifold is related to the equation:

(7) $$\lambda =k_{AB}f\left(0,B_{o}\right)-D_{A}{\vec{k}}^{2}-k_{A}$$

where the stability parameter λ can be negative or positive (Figure 1). This manifold promotes instabilities of the homogeneous solution (λ>0), generating structures containing “bricks” A.

For a finite system of size L, at the spatial dimension d=3 and from Equation (7), the explicit condition for structure formations λ>0 becomes:

(8) $$k_{AB}f\left(0,B_{o}\right)-k_{A}>D_{A}\frac{\alpha }{L^{2}}\left(n^{2}+l^{2}+m^{2}\right)$$

for a set of integers $\left(n,l,m\right)\propto L\vec{k}$ different from zero. Additionally, in the above equation, $\alpha \sim \pi^{2}$ depends explicitly on the type of boundary condition.

Equation (8), concerning the generation of new structures from A=0, needs to be considered in terms of the following points:

- Equation (5) is necessary, but not sufficient, to satisfy Equation (8).
- New structures are favored for smaller values of the set (n,l,m), i.e., simple patterns. Tangentially, it is noted that simple patterns progress in the early stage of development of an embryo [11].
- The generalization of Equation (8) to small spatial dimensions of one or two is direct (formally, l≡m≡0 or l≡0, respectively). In the next sections, results are obtained for any spatial dimension d, including fractional dimensions.
- The generation of structures requires a minimal spatial size (see Equation (11)), as follows:
  (9) $$L_{c}{}^{2}=D_{A}\alpha /\left(k_{AB}f\left(0,B_{o}\right)-k_{A}\right)$$
  where the parameter $L_{c}$ characterizes the cell size.

For practical purposes, it is convenient to write the stability parameter λ in Equation (7) in terms of the basic cell size $L_{c}$:

(10) $$\;\lambda =\frac{D_{A}\alpha }{L_{c}{}^{2}}\left(1-\frac{L_{c}{}^{2}}{L^{2}}\left(n^{2}+l^{2}+m^{2}\right)\right)$$

Equation (8), for the generation of structures, is straightforwardly reformulated as:

(11) $$L^{2}>L_{c}{}^{2}\left(n^{2}+l^{2}+m^{2}\right)$$

Note that Equation (10) formally generates a sphere in the (n,l,m) space of dimension three.

The work thus far has been mostly concerned with the physics and chemistry aspects of the patterns. The next section incorporates the concept of heredity discussed in Section 1.

## 5. Number of Equivalent Structures and Heredity

Temporal continuity, or memory, is, in principle, intrinsically related to reaction–diffusion equations, such as Equations (1) and (2), where the initial conditions formally define the time evolution. However, heredity is not only connected to the continuous history of a system, but is also related to the formation of new structures, diversity, and viability. In this sense, I proceed, as in statistical mechanics, by adopting the notion of equivalent configurations [12,13,14,15].

We are locking, according to pattern diversity, in such a way that some equivalent structures, eventually, are not eliminated (viability) in open systems. This process of selection can include intraspecific competition [33] for chemical resources or “depredation” [34].

As in statistical mechanics, for the set of integers (n,l,m) and fixed λ, Equation (10) defines a variety of equivalent structures in d=3. Accordingly, for any spatial dimension d, assuming that the number $N_{H}$ of equivalent structures (diversity) is proportional to the area of the surface of a hyper-sphere [12,13,14,15], we have:

(12) NH=12d2πd/2Γ(d2)(LLc)d−1{1−λLc2DAα}(d−1)/2

where d, as previously mentioned, is the dimension of the physical space and Γ is the gamma function.

In the case of a physical interface, such as a smooth rock surface covered by slime [8,11], d=2 is appropriate. Moreover, in Equation (12), d can be considered as characterizing a fractal dimension [35,36,37,38,39,40,41]. Note that the function πx/2/Γ(x2), for 1<x<3, ranges approximately between one and six.

As long as λ>0, from Equation (12), the maximal $N_{H}$ of equivalent structures is given by:

(13) NH,max=12d2πd/2Γ(d2)(LLc)d−1

Moreover, this is useful in making estimations (next section). Concerning the concept of heredity, a large number of equivalent structures $N_{H}$ promote diversity, and, consequently, viability faces selection processes.

Finally, I briefly consider the physical substrate, where a variety of structures can develop. External constraints on a given material can generate cracks under appropriate stress conditions [42], e.g., cracks in geological rock formations [43,44,45,46]. These cracks produce $N_{H}$ enclosed regions or patches, analogous to those of mud cracks, with an average size L depending on the material and stress [47,48,49]. Each patch can be assumed as a physical region, a substrate, where a proto-tissue can eventually develop.

## 6. Time-Formation for Proto-Tissues: Role of Dimension

The following parameter values are used in making estimations:

(a) $\alpha D_{A}\sim 10^{-10}$ m^2^/s corresponding to biological molecules, such as ribonucleic acid (RNA) [6,8] at 25 °C. Note that RNA was suggested for the initial genetic basis and catalysis in primitive cells [4,11,50].
(b) A protocell of size $L_{c}\sim 10^{-5}$ m (i.e., 10 μm) and a membrane with thickness $\Delta x_{B}\sim 10^{-8}$ m.
(c) As an estimation, $N_{H}$ is assumed to be approximately equal to the number of cells in the proto-tissue.

At an arbitrary spatial dimension d, the hyper-volume $\Delta V_{B}$ surrounding a protocell of size $L_{c}$ can be estimated using the expression $\Delta V_{B}=\left(L^{d}/N_{H}\right)-L_{c}{}^{d}$. According to Equation (12), at the first order on the parameter λ, the thickness $\Delta x_{B}={\left(\Delta V_{B}\right)}^{1/d}$ becomes:

(14) $$\Delta x_{B}\sim L_{c}{\left(\lambda dL_{c}{}^{2}/2\alpha D_{A}\right)}^{1/d}$$

Using the values previously mentioned and Equation (14), the characteristic time: τ=1/λ for the structure formation as a function of dimension d is given by:

(15) $$\tau \sim 1.6d\times 10^{3d-8}\;\mathrm{years}$$

**Figure 2:**
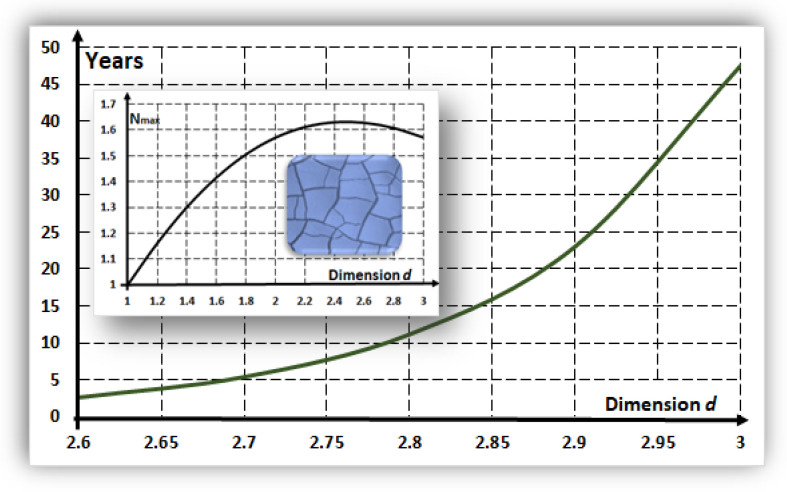
The main graph presents the formation time τ for tissues as a function of the spatial dimension d. A large spatial dimension requires a significant amount of time for proto-tissues to develop. The graph was constructed using Equation (15) and relates to cell parameters and the RNA diffusion coefficient. The inset graph shows the maximal number of equivalent structures $N_{H,max}/{\left(L/L_{c}\right)}^{d-1}$ as a function of the spatial dimension. Consequently, the maximal diversity occurs at dimension ~2.5. The illustrative blue inset picture depicts $N_{H}\sim 30$ crack patches in which hypothetical tissues can eventually develop.

The main graph in Figure 2 shows τ as a function of the spatial dimension d. Consequently, structures with a small spatial dimension, such as slime on a rock surface, develop in a few years. In contrast, structures in three dimensions (such as a liquid bubble) require decades to develop. The inset graph shows the maximal number of equivalent structures (Equation (13)) per unit of dimensionless surface, $N_{H,max}/{\left(L/L_{c}\right)}^{d-1}$, as a function of the spatial dimension d. The maximum is at the fractional spatial dimension d~2.5, a welcome consequence for the origin of life on mineral surfaces [51] and related to the maximal diversity promoting viability. The inner blue figure shows, for illustrative purposes, an ensemble of equivalent crack structures with $N_{H}\sim 30$ patches obtained by the author in a drying experiment.

## 7. Environmental Fluctuations and Optimal Dimension: Configuration Entropy

If $N_{H}$ represents the number of equivalent configurations, as occur in statistical mechanics for a macro-state [12,13,14,15], the configuration entropy of proto-tissues can be defined as follows:

(16) $$S=k_{B}ln\left(\frac{N_{H}}{{\left(L/L_{c}\right)}^{d-1}}\right).$$

In the above expression, the number of configurations is divided by the dimensionless surface to avoid the equivalent of Gibbs’ paradox 12. The Boltzmann’s constant $k_{B}$ is included to make evaluations relating to extreme environments, such as, for instance, the Atacama Desert.

**Figure 3:**
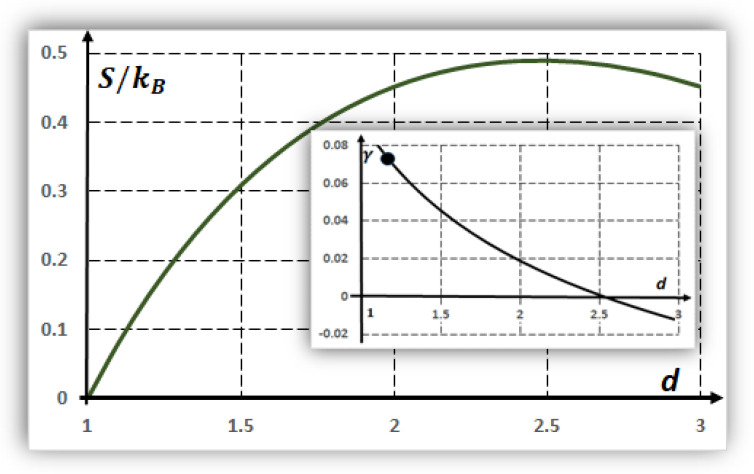
Main curve, the configuration entropy $S/k_{B}$ of proto-tissues as a function of the spatial dimension d. The maximum occurs numerically at d~2.5 and promotes diversity and complexity. Inset curve, the derivative $\gamma =\left(1/k_{B}\right)\partial S/\partial d$ defines equilibrium in a system with uncertain (fluctuating) spatial dimension of average d. At d~5, γ~0 is related to weak thermal fluctuations (γ~δT/2T) and is optimal for proto-tissue formation. The black point corresponds to γ≈0.076 for the Atacama Desert (Chile).

Figure 3 shows the dimensionless entropy function $S/k_{B}$ and its derivative (inset):

(17) $$\;\gamma =\left(1/k_{B}\right)\partial S/\partial d,$$

as a function of the spatial dimension d. The entropy Equation (16) has a maximum at the dimension d~2.5. The quantity γ, analogous to β in thermodynamics [12,13,14,15], corresponds to a parameter-defining equilibrium when the spatial dimension is fluctuating [52].

According to the equipartition principle in thermodynamics [12,13,14,15], the entropy is $\delta S=C_{V}\delta T/T,$ where T is the temperature and $C_{V}$ is the specific heat. Then, from Equation (17), a theoretical estimation for the primitive environment stays, as follows:

(18) $$\gamma \sim \frac{1}{2}\frac{\delta T}{T}.$$.

Accordingly, the following results are obtained from Equation (18) and the inset curve (i.e., γ) in Figure 3:

(a) If the spatial dimension is d~2.5, then γ~0 and weak fluctuations, δT/T~0, exist around this fractional dimension. These results indicate stable refuges against fluctuations. This point is fully complementary with a maximal diversity of structures when d~2.5 (Section 6).
(b) In the same way, for a spatial dimension of d ~ 2.0, i.e., a smooth slime sheet, |δT/T|~0.04, corresponding to environmental variations. Additionally, for d~3, e.g., a bubble in a liquid medium, the thermal variations are |δT/T|~0.02.
(c) As a geologic example, in the Atacama Desert of Chile, rock temperatures vary between approximately [53] 0 and 45 °C. If the average temperatures are in the order of 296 oK, then $\gamma_{env}=\frac{\delta T}{2T}=\frac{45}{2\times 296}\sim 0.076$ corresponds to a spatial dimension smaller than two for the formation of hypothetical proto-tissues in these extreme conditions.

In summary, a spatial dimension of d~2.5 promotes weak thermal fluctuations for proto-tissues, and there is, similarly, maximal diversity (Section 6).

## 8. Conclusions

The stability rate λ defines a characteristic formation time for proto-tissues (τ ~ 1/λ). The estimation of this characteristic time as a function of the spatial dimension was obtained (Figure 2). The estimate ranges from a few years for surfaces near a dimension of two (e.g., slime) to decades for three-dimensional environments (e.g., possibly liquids). These estimates were made using acceptable data from cells and the RNA diffusion coefficient.

When variations in the thermodynamic primitive environment are considered, from stability arguments, physical substrates containing proto-tissues with an integer spatial dimension of two or three are associated with thermal fluctuations. In contrast, for fractional dimensions at approximately 2.5, the substrate prevents hard thermal fluctuations (refuge), and there is maximal diversity, promoting replication, variation, and heredity.

As stated in the introduction, the question of “what is life?” were not touched upon during this work and are difficult to answer. Nevertheless, these ultimate questions were the motivation for the present work. Interesting efforts, some of which are controversial [54,55,56,57], have been made to answer these questions [58,59,60,61]. Furthermore, a number of studies have been captivatingly curious [62,63], including inorganic trashes “fingerprint” [64], interesting sharpness [65], and revising complexity for amino acids [66].

Finally, several groups are investigating protocell aggregates [7,8,9,10,11,54,55,56,57,58,59,60,61,62,63,64,65,66,67]. Interesting open questions are related to this and can be inquired about in reference to Damer and Damer [67]. Additionally, regarding living systems, Maturana and Varela propose that they are cognitive systems [68]. No separation exists between both concepts.

## Data Availability

Not Applicable.
